# Genetic Overlap Between DSM-IV Major Depressive Disorder and Suicidal Behaviors: Evidence from Polygenic Risk Scores in Young Adult Twins

**DOI:** 10.1007/s10519-025-10234-0

**Published:** 2025-09-24

**Authors:** Nathan A. Gillespie, Mei-Hsin Su, Séverine Lannoy, Mallory Stephenson, Miguel E. Rentería, Zuriel Ceja, Ian B. Hickie, Nicholas G. Martin, Alexis C. Edwards

**Affiliations:** 1 Virginia Institute for Psychiatric and Behavioral Genetics, Department of Psychiatry, Virginia Commonwealth University, Richmond, VA 23298 USA; 2 QIMR Berghofer Medical Research Institute, Brisbane, QLD 4006 Australia; 3 Brain & Mental Health Program, QIMR Berghofer Medical Research Institute, Brisbane, QLD 4006 Australia; 4 School of Biomedical Sciences, Faculty of Medicine, The University of Queensland, Brisbane, QLD 4072 Australia; 5 School of Biomedical Sciences, Faculty of Health, Queensland University of Technology, Brisbane, QLD Australia; 6 Brain and Mind Institute, University of Sydney, Camperdown, NSW Australia

**Keywords:** Major depressive disorder, Suicidal behaviors, Attempt, Polygenic risk scores, Twin study, Genetics

## Abstract

Suicidal thoughts and behaviors (STBs) represent a significant public health concern. This study aimed to examine the extent to which polygenic risk scores (PRSs) for suicide attempt and major depression (MD) explain variance in suicidal ideation, plans, and attempts among young adult twins. Data from 2876 participants of European ancestry in the Brisbane Longitudinal Twin Study were analyzed. PRSs for MD and suicidal behavior (SB PRS) were calculated. Multivariate twin modeling was used to estimate genetic and environmental influences on DSM-IV Major Depressive Disorder (MDD) diagnosis and STBs, as well as their associations with PRSs. Heritability estimates were higher for STB phenotypes (51–80%) compared to DSM-IV MDD (39–41%). The MD PRS showed more consistent genetic correlations with DSM-IV MDD, while both PRSs showed modest correlations with suicide outcomes. Multivariate analyses revealed remarkably high genetic correlations among STBs (rA = 0.85–0.99) and moderate genetic correlations with MDD (rA = 0.48–0.65). Environmental factors contributing to DSM-IV MDD risk were largely distinct from those influencing suicide-related phenotypes. This study provided compelling evidence for shared genetic architecture between DSM-IV MDD and STBs. The MD PRS demonstrated more consistent prediction of MDD compared to the SB PRS, though both showed modest correlations with suicide outcomes. These results have important implications for risk assessment strategies, though the substantial unique environmental influences highlight the need to address modifiable environmental risk factors. Future research should focus on replication in larger, more diverse samples and exploring the interactions between genetic risk factors and environmental influences across the lifespan.

**Supplementary Information:**

The online version contains supplementary material available at 10.1007/s10519-025-10234-0.

## Background

Suicidal thoughts and behaviors (STBs) represent a significant public health concern, with approximately 700,000 individuals dying by suicide worldwide annually, including over 49,000 in the US (Centers for Disease Control and Prevention (CDC), 2020; World Health Organization 2021). For every death by suicide, the WHO estimates that there are 20–25 attempts (Auerbach et al. 2019). Major Depressive Disorder (MDD) is one of the leading risk factors for STBs (Nock et al. 2013), with genetic studies indicating substantial overlap between these phenotypes (Mullins et al. 2022). Despite the severity of this issue, our understanding of the genetic architecture underlying STBs remains incomplete. This study aims to address this gap by examining the extent to which polygenic risk scores (PRSs) for suicidal behaviors (SB) and major depression (MD) explain variance in measures of suicidal ideation, plans, and attempt within a sample of young adult twins with genotypic data.

The heritability of STBs has been established through twin and family studies, with estimates ranging from 0.17 to 0.55 across different outcomes (Brent and Melhem 2008; Edwards et al. 2021; Fu et al. 2002; Voracek and Loibl 2007). Recent advances in genomics have yielded promising results, with genome-wide association studies (GWAS) of suicide ideation, attempt, and death reporting SNP-based heritability estimates of h²SNP = 0.05 for suicidal ideation (Ashley-Koch et al. 2023), h²SNP = 0.07 for suicide attempt (Mullins et al. 2022), and h²SNP = 0.06 for suicide attempt (Docherty et al. 2023). For suicide death, the Docherty et al. (2020) GWAS study estimated heritability at h²SNP = 0.25, with a liability scale h²SNP estimate of 0.16.

Just as PRSs for MD have been shown to predict MDD phenotypes in independent samples with modest but significant associations (Howard et al. 2019; Mullins et al. 2022), PRSs for suicide attempt - henceforth termed suicide behavior PRS (SB PRS) - have demonstrated predictive ability for STBs in independent cohorts. For instance, studies have used suicide attempt PRSs to predict lifetime suicide attempt in U.S. army soldiers and individuals with familial risk of bipolar disorder, showing significant but modest associations (Campbell-Sills et al. 2023; Stein et al. 2024). However, the predictive power of these PRSs remains limited due to the complex genetic architecture of STBs, which likely involves multiple interacting variants and environmental factors. This complexity underscores the need to explore comorbidity with psychiatric disorders.

Significant comorbidity between STBs and other psychiatric disorders, particularly internalizing disorders like MD, is well-documented, as shown by epidemiological studies (Auerbach et al. 2019; Basterfield et al. 2024; Borges et al. 2010; Olfson et al. 2017), and further supported by genetic correlations between suicide attempt and psychiatric disorders (Docherty et al. 2023; Mullins et al. 2022). However, a significant proportion of individuals who engage in STBs do not have a history of psychiatric problems (Oquendo et al. 2024), highlighting the need to explore non-clinical or subclinical characteristics as potential risk factors. This genetic overlap with psychiatric disorders suggests that molecular genetic tools may extend beyond within-phenotype prediction. Indeed, Goto et al. (2016) and Lee et al. (2025) have recently shown that PRSs for psychiatric disorders (including depression, bipolar disorder, and post-traumatic stress disorder) exhibited modest but significant associations with STBs, indicating cross-phenotype predictive potential.

Despite these insights, a critical gap persists in the literature regarding genetically informative within-person studies that can disentangle the unique genetic influences on STBs from those shared with comorbid conditions. While prior twin studies have examined genetic influences on individual STBs (Brent and Melhem 2008; Fu et al. 2002; Voracek and Loibl 2007), the genetic architecture linking MDD, suicide ideation, planning and attempt remains unexplored. Notably, no study has employed multivariate twin models that incorporate PRSs for MD or STBs to determine whether or not these phenotypes share common genetic factors or have distinct genetic etiologies. Understanding these relationships is crucial for identifying shared vs. unique pathways to suicide risk, motivating the current investigation.

By combining genetically informative twin methods with molecular genetic data derived from GWAS summary statistics, we can gain insights beyond those achievable through either approach alone. While twin analyses can estimate the genetic correlations between depression and STBs, incorporating PRSs allows us to examine how well current molecular genetic predictors capture these shared genetic influences. Specifically, our analyses aim to estimate: (1) the contribution of both the MD PRS and MDD diagnosis to each of the three STB phenotypes, evaluating its predictive power for both MDD and STB phenotypes; and (2) the contribution of both the SB PRS and MDD diagnosis to each of the three STB phenotypes, determining whether genetic risk for suicide attempt is specific to STBs or also influences MDD. We hypothesize that the MD and SB PRSs will explain small but significant amounts of variation in the self-report STBs, with the SB PRS potentially capturing molecular risk factors distinct from those accounted for by MD PRSs. Additionally, by applying multivariate twin modeling, we will determine the extent to which suicide ideation, planning and attempt share common genetic and environmental influences while also testing whether or not genetic factors influencing these STBs are distinct from those impacting an MDD diagnosis. This combined methodological approach offers unique insights into the genetic architecture of suicide risk, improves our understanding of the etiology of suicidal behavior, and informs the development of more targeted prevention efforts.

## Methods

### Subjects

Data for this study come from the ongoing Brisbane Longitudinal Twin Study (BLTS). The BLTS was launched in 1992 to study melanocytic nevi and comprises > 7,000 young adult twins, siblings and parents of European ancestry with longitudinal assessments when twins were aged 12, 14, 16, 21 and 25 years (Couvy-Duchesne et al. 2018; Gillespie et al. 2013; Wright and Martin 2004). Specifically, we used GWAS summary statistics from Howard et al. (2019) and Docherty et al. (2023) to compute PRSs for major depression (MD) and suicide attempt (SA), respectively, in the BLTS ‘19Up’ sample of 2,876 individuals (2,142 monozygotic and dizygotic twins, including unlike-sex DZ pairs, and 734 non-twin siblings; 67% female, mean age = 25.9 years, SD = 3.6, range = 19–39), which also provided phenotypic data on MDD and SA (Wright and Martin 2004). For DZ twins, assignment as twin 1 or twin 2 was random and not based on sex.

### Ethics

All BLTS assessment protocols were approved by the QIMR Berghofer Medical Research Institute-Human Research Ethics Committee. Written informed consent was obtained from all subjects.

### Measures

#### Self-report Suicidal Behavior

As part of the survey assessing demographic, general, and mental health items, subjects who endorsed having ever felt ‘depressed or down’, or ‘sad, blue, low, or discouraged’ for ‘most of the day and nearly every day for two weeks or more’ were asked additional questions. These questions, in the context of DSM-IV clinical criteria for assessing MDD, inquired if ‘during that period of time’ they had ‘on more than one occasion’: (i) thought about taking their own lives (suicide ideation); (ii) planned to take their own lives (suicide plans); and (iii) tried to take their own lives (suicide attempt). Each response was recorded as a binary outcome (yes/no). While we refer to ideation, plans, and attempts collectively as STBs throughout, it is important to note that only attempts constitute actual suicidal behavior, whereas ideation and plans represent thoughts and intentions.

#### Genome-Wide Association Study (GWAS) Data

The BLTS GWAS data collection, including a description of SNP quality control, alignment, ancestry checks, and imputation methods are described in detail elsewhere (Couvy-Duchesne et al. 2018; Gillespie et al. 2013; Wright and Martin 2004). Briefly, sample SNP quality control relied on standard QC protocols (Purcell et al. 2007): (i) sex check; (ii) use of samples with ≥ 95% genotyping call rate; (iii) heterozygosity (Plink F) values ranging − 0.10 < F < 0.10; (iv) accuracy of familial relations based on IBD sharing; (v) HWE p-value of the two parental founders should be ≥ 0.001; and (vi) a Mendelian error rate < 1%. Post-SNP QC data were aligned to the Human Reference Consortium (HRC) 1.1, or the 1000 Genomes reference panel (both on Human Genome build 37 HG19) using the following tools: (i) Lift-over, to lift the SNP data from one build to another; and (ii) the HRC or 1000G Imputation preparation and checking tool. We compare the chosen reference panel with the input SNP data by the following: equal name; chromosome; base-pair location and alleles for the SNP as outlined in the McCarthy’s group tool (https://www.well.ox.ac.uk/~wrayner/tools/). If mismatching alleles existed, the inversion of the DNA strand was checked and a SNP-list to align the data to the positive strand was created. The software tests if SNP MAFs are similar to the expected MAFs of the reference panel (+/−0.20) before listing problematic SNPs. Once aligned, a principal component analysis was performed on the combined data to identify ancestral outliers in Australia. Outlier removal depended on the deviation of the module eigenvector scores (Martin et al. 2017). Once outliers were removed, allele frequencies were again compared with the reference panel. In order to analyse the largest possible sample and to avoid stratification due to missing or non-missing SNPs over platforms and studies, all data were imputed to 1000 Genomes or the HRC reference panel on the Michigan imputation server (Das et al. 2016). After imputation, best-guess SNP genotypes were generated using the output VCF files. Post-imputation SNP QC was then used to inspect MAFs, to apply a stringent HWE filter *p* < 0.001, and to identify and remove Mendelian errors with additional filtering based on a SNP imputation quality R^2^ > 0.60–0.90. After imputation, the BLTS Genotypic data had 7,917,029 QC’d SNPs with MAF ≥ 1% and R^2^ ≥ 0.3.

#### Polygenic Risk Score (PRS) Estimation

PRSs for MD and suicidal behavior (SB) were based on the GWAS summary statistics provided by Howard et al. (2019) and Docherty et al. (2023) respectively. The MD PRS, based on a meta-analysis of 246,363 cases and 561,190 controls of European descent, reflects a heterogeneous construct including both clinical diagnoses and non-clinical, self-reported depressive symptoms, distinct from the DSM-IV Major Depressive Disorder (MDD) phenotype assessed in this study, which adheres to strict diagnostic criteria. The SB PRS, computed from a GWAS of 43,871 suicide attempt cases (including a small proportion of suicide deaths) and 915,025 ancestry-matched controls using a leave-Brisbane-out approach to avoid overlap with BLTS data, focuses primarily on suicide attempt. We, therefore, use ‘SB PRS’ to refer to this narrow GWAS construct, whereas our STB phenotypes encompass the broader spectrum of suicidal ideation, planning, and attempt. Both PRSs were estimated using the PLINK software version 1.90 (https://www.cog-genomics.org/plink2) (Chang et al. 2015) using the Polygenic Risk Score Continuous Shrinkage (PRC-CS) approach (Ge et al. 2019). PRS estimates were based on SNP effect sizes from all reported SNPs, derived from European population linkage disequilibrium information in the 1000 Genomes reference set. Prior to analyses, the MD and SB PRSs were standardized to a mean of zero and a standard deviation of one. Principal components were not regressed out of the PRS calculations as population stratification is controlled in twin designs, where siblings serve as perfectly matched controls for ancestry.

#### Statistical Analyses

The OpenMx 2.20.6 software package (Boker et al. 2011) in R 4.2.2 (R Development Core Team 2018) with the NPSOL optimizer (Zahery et al. 2017) was used to test basic assumptions of threshold homogeneity (within twin pairs and across zygosity), calculate phenotypic and twin pair correlations (along with 95% confidence intervals), followed by multivariate twin modelling (Neale and Cardon 1992).

### Mean and Variance Homogeneity

Each observed phenotypic variable is assessed as four distinct measurements: MZ twin 1, MZ twin 2, DZ twin 1, and DZ twin 2. Models for twin data usually predict that the means and variances are the same across all (four) instances. Therefore, we began by testing these predictions of (i) equal means and (ii) equal variances across twin 1 and twin 2 within each zygosity group. These tests were followed by tests for mean and variance equality across zygosity.

### Phenotypic and Twin Pair Correlations

Prior to our model fitting we estimated phenotypic correlations between the suicide ideation (SI), suicide plan (SP), and suicide attempt (SA) items, the DSM-IV MDD diagnoses, the PRSs for MD and SB, and the covariates of sex and age at assessment. We also estimated twin pair correlations by zygosity for SI, SP, and SA (based on the threshold liability model – see Supplement).

If familial aggregation in a complex trait exists and is entirely attributable to shared family environments, then two expectations should hold: (1) both MZ and DZ twin pair correlations are statistically significant and greater than zero; and (2) the MZ and DZ twin pair correlations should not be significantly different from each other. At any nominal significance level alpha, these tests should not be found significant at more than the nominal rate. We note that if both correlations are not significantly different from zero, this would suggest a lack of familial aggregation altogether, rather than aggregation stemming from shared environmental factors.

### Sex Differences

We also conducted exploratory analyses of sex differences in the endorsement rates of MDD and suicide-related phenotypes. For these 2 × 2 contingency tables (sex by diagnosis), we used Pearson’s chi-squared tests with Yates’ continuity correction (Yates 1934). By reducing the chi-square value, the Yates’ correction provides a more conservative test, helping to prevent Type I errors that can occur when analyzing discrete data, particularly with smaller cell frequencies. We also calculated Cramer’s V, which ranges from 0 to 1 and provides a measure of effect size for categorical variables that is independent of sample size. Values of 0.1, 0.3, and 0.5 represent small, medium, and large effect sizes respectively (Cohen 1988).

### Multivariate Analyses

We applied the Classical Twin Design (CTD) to decompose the total variation in each suicide ideation, plan, and attempt into additive (A) genetic variance, shared or common environmental (C), and non-shared or unique (E) environmental variance components (see Supplementary Figure S1). This decomposition is achieved by exploiting the expected genetic correlations between MZ and DZ twin pairs; MZ twin pairs are genetically identical, whereas DZ twin pairs share, on average, only half of their genes. Therefore, MZ and DZ twin pair correlations (rA) for additive Genetic effects are fixed to 1.0 and 0.5 respectively. The CTD assumes neither genotype by environmental interactions nor genotype-environment correlations, and that parental mating is random. It also assumes that shared environmental effects are equal for MZ and DZ twin pairs, i.e., equality of parental treatment, equality of environmental exposure, and no effects caused by placentation (Scarr 1968). Given this equal environmental assumption, the MZ and DZ twin correlations (rC) for shared or common (C) environmental influences are both fixed to 1.0. Since all non-shared environmental influences (E), which include measurement error, are by definition uncorrelated, the MZ and DZ twin pair correlation (rE) for these ‘E’ influences is fixed to zero.

We did not model genetic non-additivity or dominance (D). In the CTD, the ‘C’ and ‘D’ influences are negatively confounded, and therefore, cannot be modelled simultaneously (Martin et al. 1978). Since the sample sizes required to detect ‘D’ as a source of variation are very large, even for variables measured on a continuous liability scale, we chose to model ‘C’ influences in all subsequent univariate and multivariate models. Finally, because the STBs were assessed on a population-based sample of twins, the estimates of A, C, and E are assumed to capture variation arising from both risk and protective factors.

**Fig. 1:**
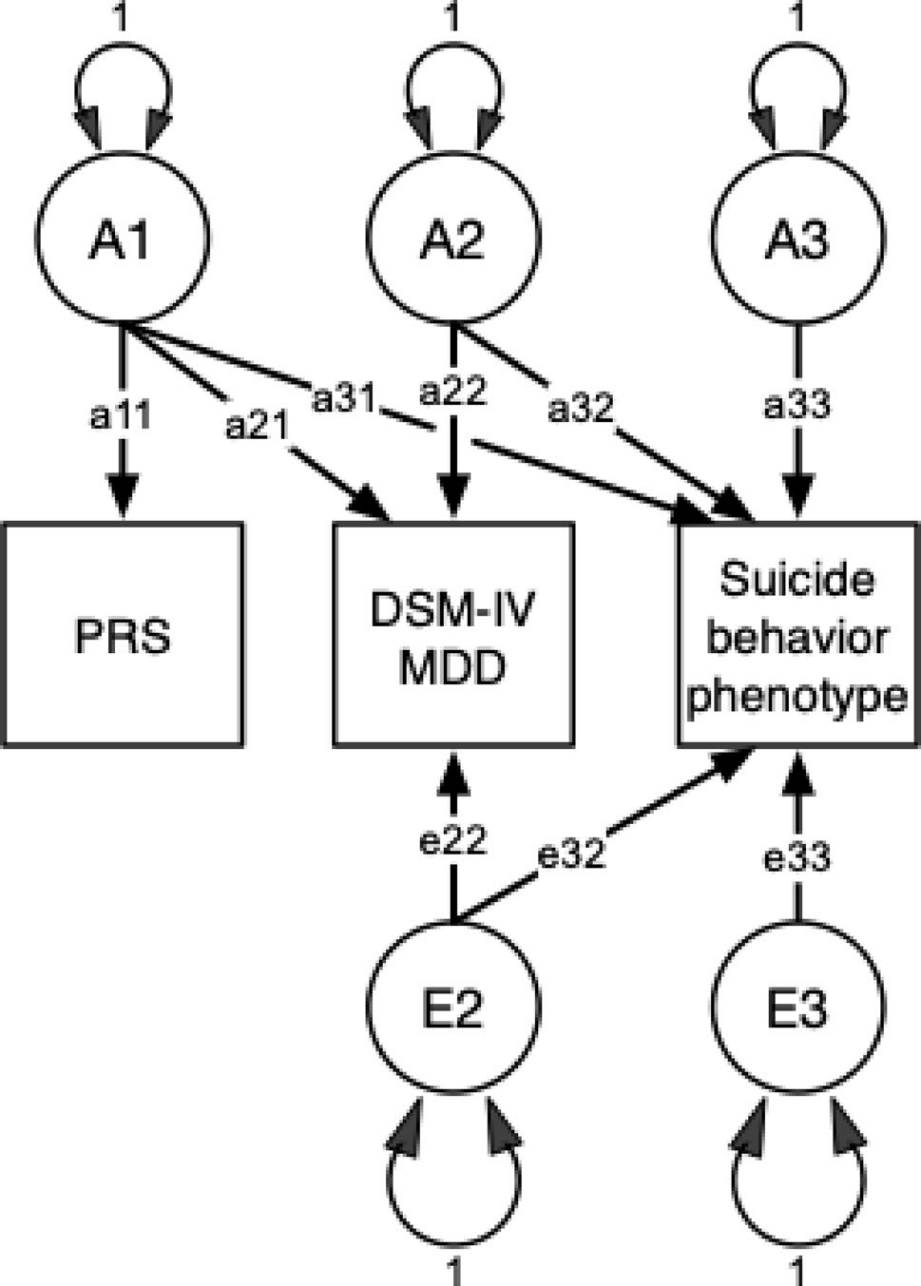
Theoretical illustration of a Multivariate Cholesky Decomposition showing genetic and environmental pathways between polygenic risk scores, DSM-IV MDD, and suicide behaviors. *PRS* polygenic risk score, *DSM-IV MDD* diagnostic and statistical manual of mental disorders, 4th Edition Major Depressive Disorder. A1, A2, and A3 denote additive genetic sources of variance for the PRS, MDD-IV MDD diagnosis, and the suicide behavior phenotype respectively. E2 and E3 denote non-shared environmental sources. Shared environmental influences (C) and latent variable means not illustrated for brevity. The PRS variance is entirely attributable to additive genetic variance (i.e., no E1 or C1). Single-headed arrows denote pathway coefficients from latent to observed variables. Double-headed arrows indicate fixed unit variance for each latent source

A multivariate extension of this standard univariate approach was necessary to model accurately the complex relationships between each of the PRSs, the DSM-IV MDD diagnosis, and the STBs phenotypes (see Fig. 1). To account for the conditional nature of the data collection, where the STBs were assessed only in the context of DSM-IV clinical criteria for MDD (as described elsewhere in the methods), we used a trivariate Cholesky decomposition to estimate simultaneously the genetic and environmental influences on the PRS, MDD, and each STB phenotype. This trivariate approach captures the covariance between the PRS, MDD, and STBs, including the contribution of the PRS to the genetic variance of both MDD and STB phenotypes. Specifically, our analyses aimed to estimate:

1. The contribution of both the MD PRS and MDD diagnosis to each of the three STB phenotypes. This allows us to assess the predictive power of genetic risk for MD on both the MD and suicidal behavior phenotypes.
2. The contribution of both the SB PRS and the MDD diagnosis to each of the three STB phenotypes. This helps us to understand whether or not genetic risk for suicide attempt is specific to STBs or also influences MDD.

To model these relationships, we employed a multivariate ‘ACE’ Cholesky Decomposition (Fig. 1), a method based on the matrix factorization technique originally developed by André-Louis Cholesky (Cholesky 1910) and later adapted for behavioral genetic analyses (Neale and Cardon 1992). In this approach, the first observed phenotype (PRS) is assumed to be influenced by a latent factor (A1) that may also affect subsequent variables (DSM-IV MDD and suicide item). Each additional variable is assumed to be influenced by new latent factors (e.g., A2 for DSM-IV MDD) that may also impact subsequent variables. We specified separate Cholesky Decompositions for additive genetic (A), shared environmental (C), and individual-specific environmental (E) sources of variance. This method allows us to estimate the relative contributions of genetic and environmental influences both within and between phenotypes. By analyzing the resulting patterns of genetic and environmental correlations, along with PRS contributions, we can elucidate shared and unique etiological factors underlying the liability to MDD and STBs.

### Model Fit

For each multivariate analysis, we employed a stepwise approach to determine the best-fitting model. First, we assessed the significance of each of the A, C and E parameters using the change in the minus two Log-Likelihood (Δ−2LL), which under certain regularity conditions is asymptotically distributed as chi-squared with degrees of freedom equal to the difference in the number of free parameters in the two models. We also used Akaike’s Information Criterion (AIC) (Akaike 1987) to balance model complexity and explanatory power in determining the best-fitting model.

In evaluating model fit, we tested reduced models (e.g., CE models) by dropping specific genetic pathways (a21, a31, a22, a32, a33), which corresponds to a 5-degree-of-freedom (df) test, reflecting the elimination of (i) genetic covariances between the PRS and both the DSM-IV MDD diagnosis and the STB phenotype, and (ii) the genetic variance-covariance structure between the DSM-IV MDD diagnosis and the STB phenotype. The genetic pathway from A1 to PRS (a11) was always retained, as it represents the direct genetic contribution to the PRS measure (see Fig. 1). This approach was used to evaluate:

1. The genetic pathways from each PRS to MDD.
2. The genetic pathways from the PRSs to each STB, and.
3. The genetic and environmental pathways from MDD to each of STB.

By using the 95% CIs, we could determine the statistical significance of these pathways while maintaining the overall structure of the best-fitting model. This method allowed us to evaluate the importance of genetic and environmental influences between the PRSs, MDD and STBs.

## Results

Supplementary Table S1 shows the numbers of complete and incomplete twin pairs by zygosity for each measure. For the suicide ideation, suicide plan, and suicide attempt items, the sample consisted of 148 complete twin pairs (64 MZ, 84 DZ) and 453 singletons. The DSM-IV MDD diagnosis had a larger sample, comprising 901 complete pairs (383 MZ, 518 DZ) and 350 singletons.

Supplementary Table S2 shows endorsement rates for each phenotype across twin pairs and zygosity. Among individuals meeting DSM-IV MDD criteria (16.7–19.7% of the sample), suicide ideation has the highest endorsement rate, ranging from 34.5 to 41.9% across all twin groups. Suicide planning is endorsed less frequently, with rates between 11.5% and 17.0%. Suicide attempts have the lowest endorsement rate, ranging from 4.7 to 8.8%. The high rates of STBs reflect that these items were assessed only in the context of DSM-IV clinical criteria for MDD.

“The majority of the subjects had both phenotypic and Genetic data. While PRSs could be calculated for anyone with Genetic data, some subjects had missing or poor-quality genetic data that prevented PRS calculation. Among subjects with a DSM-IV MDD diagnosis, 84.2% had MD PRS and 88.7% had SB PRS. For those who responded to the suicide-related phenotypes, 86.4% had MD PRS and 90.0% had SB PRS. The slight differences in PRS availability reflect variations in genetic data quality and coverage across the different GWAS summary statistics used to calculate each PRS.

### Testing the Assumption of Threshold Homogeneity

Prior to conducting twin modeling on the combined monozygotic (MZ) and dizygotic (DZ) twin data, we tested the assumption of mean and variance homogeneity for each phenotype using the residualized data. Supplementary Table S3 presents the model fitting comparisons between the fully saturated and constrained threshold homogeneity models. The Threshold Homogeneity Model, which constrains the thresholds to be equal within twin pairs and across zygosity, provides a good fit for all three STBs and the DSM-IV MDD diagnosis. Based on these results, all subsequent analyses proceeded under the assumption of threshold homogeneity for each of the three suicidal behavior items.

### Strength of Association

**Fig. 2:**
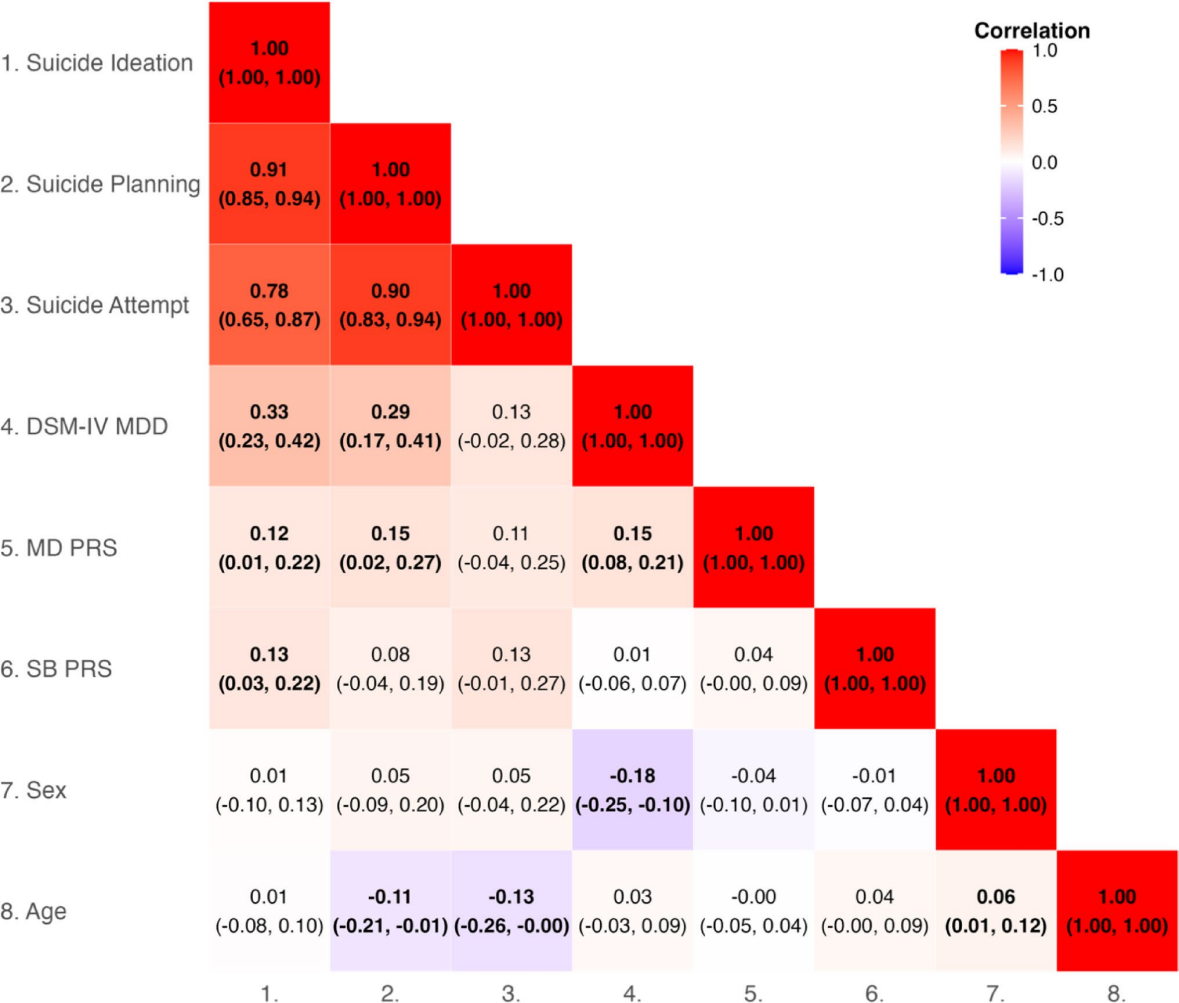
Full Information Maximum Likelihood phenotypic correlations between suicide behaviors, DSM-IV MDD, polygenic risk scores, sex and age. *MD PRS* major depression polygenic risk score, *SB PRS* suicide behavior polygenic risk score, *DSM-IV MDD* diagnostic and statistical manual of mental disorders, 4th Edition Major Depressive Disorder. Numbers in parentheses represent 95% confidence intervals. FIML is a statistical method that uses all available data from each participant, even when some data points are missing resulting in less biased estimates & greater statistical power compared to methods like listwise deletion

The phenotypic correlation heatmap in Fig. 2 reveals strong positive associations between suicide ideation, plan, and attempt (*r* = 0.78 to 0.91). While these items were assessed only among those meeting DSM-IV MDD criteria, each STB was assessed independently, without conditioning on the presence of other STBs. This high degree of interrelation therefore represents naturally occurring relationships between these behaviors. DSM-IV MDD shows moderate positive correlations with suicide ideation and planning (*r* = 0.33 and 0.29 respectively), but a weaker, non-significant correlation with suicide attempt (*r* = 0.13). This suggests that while depression is associated with suicidal thoughts, its relationship with actual suicide attempts is less pronounced in this sample.

PRSs for MDD and SB demonstrate weak but mostly significant correlations with STBs and MDD phenotypes (r ranging from 0.01 to 0.15), highlighting a modest genetic component underlying the phenotypic associations among these phenotypes.

Regarding demographic factors, we examined sex differences in the endorsement of the STBs and MDD diagnosis. Pearson’s chi-squared tests with Yates’ continuity correction revealed no statistically significant sex differences in suicide ideation [χ²(df = 1) = 0.43, *p* = 0.51], plan [χ²(df = 1) = 0.56, *p* = 0.46], and attempts [χ²(df = 1) = 0.12, *p* = 0.73], with very small effect sizes (Cramer’s V ≤ 0.027) for all three variables. These results are corroborated by the weak, non-significant correlations between sex and suicide ideation (*r* = 0.01), planning (*r* = 0.05), and attempt (*r* = 0.05) shown in Fig. 2. In contrast, there was a significant sex difference in DSM-IV MDD diagnosis [χ²(df = 1) = 18.28, *p* < 0.001], although the effect size was still small (Cramer’s V = 0.09). This finding is reflected in the negative correlation between sex and MDD (*r* = −0.18) in Fig. 2, indicating a higher prevalence of MDD in females. These results suggest that while STBs show minimal sex differences in our sample, MDD diagnosis exhibits a modest but significant association with sex.

Age-related trends in STBs and MDD were examined using logistic regression and correlation analyses (see Supplementary Figure S2). Significant negative associations were found between age and both suicide planning and suicide attempts. For every 10-year increase in age, the odds of suicide planning decreased by 43.6% (OR = 0.56, 95% CI: 0.34–0.92, *p* = 0.025), while the odds of suicide attempts decreased by 54.5% (OR = 0.45, 95% CI: 0.22–0.90, *p* = 0.029). In contrast, no significant associations were observed between age and suicide ideation or DSM-IV MDD. These findings were corroborated by the correlation heat map (Fig. 2), which showed weak negative correlations between age and suicide planning (*r* = −0.11) and attempt (*r* = −0.13), but negligible correlations with suicide ideation (*r* = 0.01) and MDD (*r* = 0.03). These results suggest that while the likelihood of suicide planning and attempts decreases with age in this sample, the prevalence of suicide ideation and a DSM-IV MDD diagnosis remains relatively stable across the age range examined.

Consequently, in our subsequent multivariate analyses, the mean liabilities for suicide planning and attempt were adjusted for the effects of age using the definition variable option in OpenMx (Boker et al. 2011). Similarly, the latent mean liability for DSM-IV MDD was adjusted for the effects of self-report biological sex. By combining data from males and females and adjusting for age and sex differences, our aim was to interpret the observed patterns of association that are shared or common across the sexes. This approach allows us to identify patterns that are generalizable across sex, and thus providing a foundation for understanding broader trends in the population.

### Twin Pair Correlations

The twin pair correlations consistently show higher concordance in monozygotic (MZ) twins compared to dizygotic (DZ) twins across all phenotypes, suggesting genetic influences on suicide ideation, plan, and attempt and DSM-IV MDD (see Table 1). Suicide attempt exhibits the strongest genetic component, with the highest MZ correlation (0.77) and the largest difference between MZ and DZ correlations. The MZ and DZ twin pair correlations for MDD are consistent with moderate heritability (MZ: 0.43, DZ: 0.12). Also, the DZ twin pair correlation point estimates for the MDD [0.54 (0.49, 0.59)] and suicide attempt [0.51 (0.45, 0.56)] PRSs are very close to the theoretically expected 0.5.

**Table 1:** Monozygotic (MZ) and dizygotic (DZ) twin pair correlations and their 95% confidence intervals for DSM-IV major depressive disorder (DSM-IV MDD) and the three suicide behaviors

Variable	Twin pair correlations	
	rMZ (95%CIs)	rDZ (95%CIs)
1. Suicide ideation	0.53 (0.20, 1.00)	0.17 (−0.16, 0.47)
2. Suicide plan	0.51 (0.03, 0.82)	0.37 (−0.01, 0.68)
3. Suicide attempt	0.72 (0.14, 0.96)	0.37 (−0.15, 0.76)
4. DSM-IV MDD	0.43 (0.25, 0.59)	0.12 (−0.07, 0.30)

*DSM-IV MDD* diagnostic and statistical manual of mental disorders, 4th Edition Major Depressive Disorder. Analyses relied on Full Information Maximum Likelihood (FIML), which uses all available data from each participant even when some data points are missing, resulting in less biased estimates and greater statistical power compared to methods like listwise deletion. Twin pair correlations calculated under the assumption of threshold homogeneity.

### Multivariate Analyses

We conducted two sets of multivariate analyses. First, we examined the covariance between each PRS (MD and SB), DSM-IV MDD, and each STB. This approach allowed us to estimate how well each PRS predicted both MDD and suicide outcomes. For both PRSs, the best-fitting models included only additive genetic (A) and non-shared environmental (E) influences (See Supplementary Tables 4 & 5).

Second, we conducted a comprehensive multivariate analysis examining the genetic and environmental architecture connecting DSM-IV MDD with all three STBs simultaneously. This analysis revealed the extent to which these phenotypes share common versus distinct genetic and environmental influences, independent of the PRS predictions.

### Part 1: Multivariate Analyses with Polygenic Risk Scores (PRSs)

In the first of six multivariate analyses examining the covariance between the MD PRS, DSM-IV MDD phenotype, and each of the STBs, the best fitting model included additive genetic (A) and non-shared environmental (E) influences only. See Supplementary Table 4 for model fitting comparisons. In the three subsequent multivariate analyses examining the covariance between the SB PRS, MDD, and each of the STBs, the best fitting models were again those that included only ‘A’ and ‘E’ influences (Supplementary Table 5). Thus, we found no evidence of shared environmental influences ‘C’ as a source of familial aggregation in suicide ideation, plans, or attempt. Note that in all six analyses, the determination of best fit was based on a non-significant change in the chi-square and the lowest Akaike’s Information Criterion (AIC). Therefore, while the CE model showed non-significant deterioration in fit for suicide planning and attempt (Supplementary Table 5), the chi-square changes for these models approached significance, whereas those for the AE models did not, supporting the AE model as the preferred fit across all cases.

The relative contributions of the ‘A’ and ‘E’ influences in each multivariate model are summarized in Table 2. The table reveals consistent patterns across all six models. For DSM-IV MDD, the additive Genetic influences range from 0.39 to 0.41, while non-shared environmental influences range from 0.59 to 0.61. In contrast, the STBs show higher and more variable Genetic influences. Suicide ideation has additive Genetic estimates ranging from 0.51 to 0.53 and non-shared environmental estimates ranging from 0.47 to 0.49. Suicide planning shows increased genetic influence (A: 0.59–0.62) and decreased environmental influence (E: 0.38–0.41), whereas suicide attempt demonstrates the highest genetic (A: 0.80) and lowest environmental (E: 0.20) influences across all models. Notably, the confidence intervals for the STBs are wider than those for MDD, suggesting much less precision in these estimates. The patterns are consistent regardless of whether or not MD PRS or SB PRS is included in the model.

**Table 2:** Standardized estimates of additive genetic (A) and non-shared environmental (E) influences with 95% confidence intervals from multivariate models of major depression PRS, suicidal behavior PRS, DSM-IV MDD, and the three suicide behaviors

	DSM-IV MDD		Suicide items	
	A (95%CIs)	E (95%CIs)	A (95%CIs)	E (95%CIs)
1. MD PRS, DSM-IV MDD & Suicide ideation	0.40 (0.24, 0.55)	0.60 (0.45, 0.76)	0.51 (0.22, 0.75)	0.49 (0.25, 0.78)
2. MD PRS, DSM-IV MDD & Suicide planning	0.41 (0.24, 0.56)	0.59 (0.44, 0.76)	0.59 (0.19, 0.85)	0.41 (0.15, 0.81)
3. MD PRS, DSM-IV MDD & Suicide attempt	0.41 (0.25, 0.56)	0.59 (0.44, 0.75)	0.80 (0.33, 0.96)	0.20 (0.04, 0.67)
4. SB PRS, DSM-IV MDD & Suicide ideation	0.39 (0.22, 0.54)	0.61 (0.46, 0.78)	0.53 (0.24, 0.76)	0.47 (0.24, 0.76)
5. SB PRS, DSM-IV MDD & Suicide planning	0.40 (0.23, 0.55)	0.60 (0.45, 0.77)	0.62 (0.25, 0.86)	0.38 (0.14, 0.75)
6. SB PRS, DSM-IV MDD & Suicide attempt	0.40 (0.24, 0.55)	0.60 (0.45, 0.76)	0.80 (0.34, 0.96)	0.20 (0.04, 0.66)

*MD PRS* major depression polygenic risk score, *SB PRS* suicidal behavior polygenic risk score, *DSM-IV MDD* diagnostic and statistical manual of mental disorders, 4th Edition Major Depressive Disorder. All models include threshold adjustments for sex effects on MDD and age effects on suicide planning and attempt, calculated under the assumption of threshold homogeneity.

As a post-hoc analysis, we performed a non-parametric bootstrap (1000 iterations) of our best-fitting bivariate AE model of MDD and suicide attempt to assess the robustness of heritability estimates for MDD and suicide attempt. Bootstrap analyses yielded heritability estimates of 40% for MDD (95% CI: 24–56%) and 74% for suicide attempt (95% CI: 20–98%), suggesting substantial uncertainty in the suicide attempt heritability estimate likely due to its lower prevalence in our sample (See Supplementary: Bootstrap analysis of heritability estimates).

**Fig. 3:**
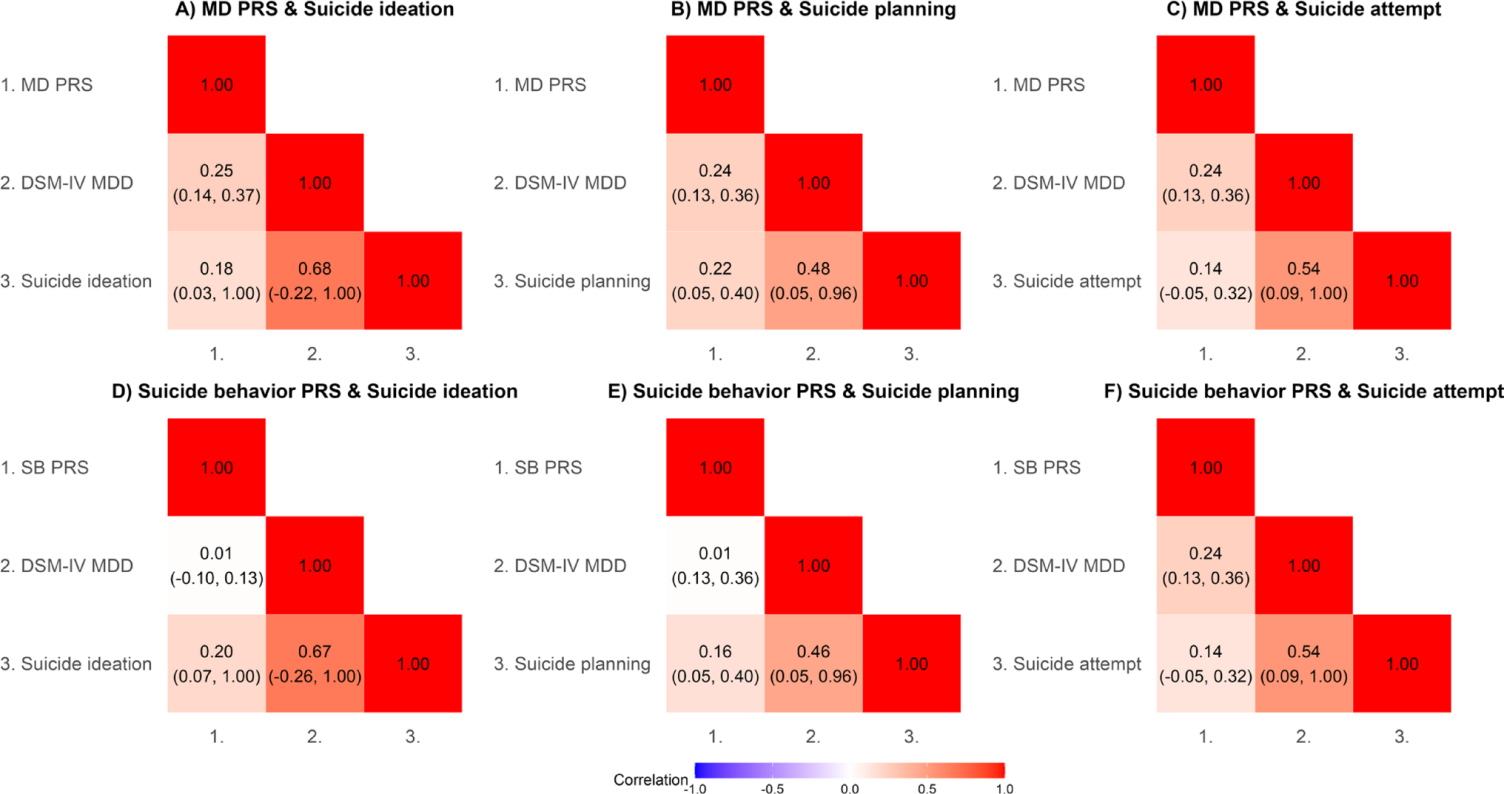
Additive genetic correlations between polygenic risk scores, DSM-IV MDD, and suicide behaviors from multivariate ‘AE’ models. *MD PRS* major depression polygenic risk score, *SB PRS* suicide behavior polygenic risk score, *DSM-IV MDD* diagnostic and statistical manual of mental disorders, 4th Edition Major Depressive Disorder. Numbers in parentheses represent 95% confidence intervals. Panels A-C show correlations with MD PRS; panels D-F show correlations with SB PRS

Figure 3 illustrates the additive Genetic correlations and their 95% confidence intervals between the DSM-IV MDD diagnosis, the three STBs (ideation, planning, and attempt), and their respective associations with the MD PRS and the SB PRS.

Key findings from these genetic correlations include:

The MD PRS showed modest significant positive additive genetic correlations with DSM-IV MDD (rA = 0.24 to 0.25), suicide ideation (rA = 0.18), and suicide planning (rA = 0.22), but a weaker, non-significant correlation with suicide attempt (rA = 0.14). The SB PRS demonstrated similar patterns, with positive genetic correlations with suicide ideation (rA = 0.20) and planning (rA = 0.16), a weaker, non-significant correlation with suicide attempt (rA = 0.14), and varying correlations with DSM-IV MDD (rA = 0.01 to 0.24). DSM-IV MDD showed moderate positive genetic correlations with suicide ideation (rA = 0.67–0.68), planning (rA = 0.46–0.48), and attempt (rA = 0.54).

These findings, based on the best-fitting AE models, suggest a shared genetic architecture among MDD and suicide-related phenotypes. While both PRSs showed modest but generally comparable genetic correlations with suicide outcomes, the MD PRS demonstrated more consistent correlations with the DSM-IV MDD diagnosis compared to the SB PRS.

The non-shared environmental correlations (rE) (Supplementary Tables S4 & S5) between DSM-IV MDD and the three STBs were generally weak and non-significant, ranging from rE = 0.07–0.21 for ideation and planning, to negative and non-significant correlations for attempt (rE = −0.44 to −0.05). These weak and non-significant environmental correlations suggest that non-shared environmental factors contributing to MDD risk are largely distinct from those influencing suicide-related phenotypes.

### Part 2: Multivariate Analysis of MDD and STBs

Table 3 summarizes the multivariate twin analyses revealing high genetic correlations among the three STBs, with especially high correlations between suicide planning and attempt (rA = 0.99), and between ideation and planning (rA = 0.91). See Supplementary Table S6 for model fitting comparisons. Suicide ideation and attempt also showed very high genetic association (rA = 0.85). DSM-IV MDD demonstrated moderate genetic correlations with all three STBs, ranging from rA = 0.48 with planning to rA = 0.65 with ideation, suggesting partially distinct genetic architectures.

**Table 3:** Genetic and environmental correlations between DSM-IV major depressive disorder and suicidal behaviors from multivariate twin analysis

	1.	2.	3.	4.
1. DSM-IV MDD	1.00	*0.06 (−0.26*,* 0.33)*	*0.12 (−0.23*,* 0.48)*	*−0.36 (−0.87*,* 0.19)*
2. Suicide ideation	0.65 (0.33, 0.98)	1.00	*0.99 (0.92*,* 0.99)*	*0.75 (0.32*,* 0.96)*
3. Suicide planning	0.48 (0.16, 0.76)	0.91 (0.82, 1.00)	1.00	*0.64 (0.14*,* 0.90)*
4. Suicide attempt	0.50 (0.14, 0.82)	0.85 (0.61, 1.00)	0.99 (0.90, 1.00)	1.00

Below diagonal: Additive genetic correlations (rA). Above diagonal: Non-shared environmental correlations (rE). 95% confidence intervals in parentheses. Based on best-fitting multivariate AE model. DSM-IV MDD diagnostic and statistical manual of mental disorders, 4th Edition Major Depressive Disorder.

Non-shared environmental correlations showed a different pattern. While environmental influences were highly correlated between suicide planning and attempt (rE = 0.64), correlations were weak between MDD and STBs, ranging from rE = −0.36 for attempt to rE = 0.12 for planning. These findings indicate that environmental factors influencing MDD risk are largely distinct from those affecting STBs, despite the shared genetic architecture.

## Discussion

Our findings provide insights into the genetic etiology of depression and STBs through two complementary approaches. First, our combined twin molecular genetic analyses demonstrated the differential prediction of the PRSs, helping to quantify the extent of shared genetic influences that can be captured by current genome-wide risk indices. Second, our multivariate twin analyses revealed remarkably high genetic correlations among STBs (rA = 0.85–0.99) and moderate genetic correlations with MDD (rA = 0.48–0.65), suggesting partially distinct genetic architectures.

The heritability estimates for suicide-related phenotypes (51–80%) compared to MDD (39–41%) suggest that Genetic factors play a stronger role in STBs. This is consistent with previous twin studies; for example, a meta-analysis of twin studies of depression produced a heritability estimate of 0.37 (Sullivan et al. 2000), whereas heritability estimates for STBs are typically higher (Brent and Melhem 2008; Edwards et al. 2021; Fu et al. 2002; Voracek and Loibl 2007). At the molecular level, we found that the MD PRS showed consistent prediction of MDD (rA = 0.24–0.25) and modest prediction of STBs (rA = 0.14–0.22), while the SB PRS showed more variable prediction of MDD (rA = 0.01–0.24) and similar modest prediction of STBs (rA = 0.14–0.20). This differential pattern of prediction may reflect the heterogeneity in the GWAS discovery samples, with the MD PRS derived from cases of varying severity while our phenotype captured specific DSM-IV MDD criteria.

Our multivariate twin analyses revealed remarkably high genetic correlations among the three STBs, with especially strong correlations between planning and attempt (rA = 0.99) and between ideation and planning (rA = 0.91). Similar patterns of high but incomplete genetic associations have also been reported for suicide attempt and death by suicide (Edwards et al. 2021; Kendler et al. 2020). These high genetic correlations are not commensurate with ideation-to-action theories (O’Connor and Kirtley 2018; Van Orden et al. 2010). Indeed, the findings from Edwards (2021) and Kendler (2020) have challenged the notion of a single liability continuum. Specifically, Edwards found that while the genetic correlation between suicide attempt and suicide death was high (rA = 0.67–0.74), it was not complete, suggesting substantial shared genetic risk but also some degree of etiological distinctiveness. Moreover, Kendler directly tested a liability threshold model in which attempt and death were assumed to differ only in severity on the same underlying liability dimension, and found this model fit the data poorly. These findings indicate suicide attempt and death cannot be considered simply milder and more severe manifestations of the same diathesis. Nevertheless, there still remains scope to falsify this hypothesis using more comprehensive multivariate twin methods. Formal comparisons, for example, between common and independent pathway models, represent an important next step to distinguish between single versus multiple liability perspectives.

The moderate genetic correlations between MDD and STBs (rA = 0.48–0.65) indicate partially overlapping genetic architectures. This is commensurate with prior evidence that genetic liability to suicide-related measures cannot be entirely accounted for by propensity toward depression (Mullins et al. 2022). We, therefore, argue that it is imperative to pursue more well-powered genetic studies of STB phenotypes, which ideally should include samples assessed outside the context of psychiatric illness. Importantly, since PRSs are not yet useful for the prediction of clinical outcomes (Docherty et al. 2021), any risk assessment for suicidality also ought to include a wide range of more informative risk and protective factors.

Although the AE model was identified as the best fit based on our a priori criteria, the power to detect shared environmental (C) variance, particularly for less prevalent complex phenotypes such as suicide planning and attempt, is limited by our sample size. Substantially larger samples are required to enhance the precision of these estimates and to elucidate fully the contributions of genetic and environmental risks (Eaves 1978; Jinks and Fulker 1970; Martin et al. 1978). Despite this limitation in detecting shared environmental variance, the absence of any shared environmental influences in our analyses is consistent with previous twin studies of STBs (Brent and Melhem 2008; Fu et al. 2002; Voracek and Loibl 2007). This replicable pattern across twin studies suggests that familial aggregation of STBs appears to be entirely attributable to genetic rather than shared environmental factors.

Regarding environmental influences unshared between siblings, our analyses revealed weak and non-significant environmental correlations between MDD and STBs. This pattern suggests that non-shared environmental factors contributing to MDD risk are largely distinct from those influencing STB phenotypes. This highlights the importance of considering both genetic and environmental factors in developing comprehensive risk assessment and prevention strategies. To our knowledge, little is known regarding specific environmental exposures that differentially impact risk of depression versus suicide-related phenotypes. However, there is evidence that latent environmental influences on suicide attempt versus death are low-to-modest (rE = 0.21–0.36) (Edwards et al. 2021); that correlations are lower between MDD and suicide attempt – which can occur outside the context of depression and other psychiatric illnesses (Oquendo et al. 2024) – is perhaps unsurprising. This observation underscores the imperative to identify specific environmental exposures that differentially increase risk for suicidality versus depression.

Broadly, our findings have implications for public health. While the current work and prior studies highlight the considerable genetic component of risk for STBs, current suicide-specific polygenic scores have very limited clinical utility (Docherty et al. 2021). We speculate that the predictive power of PRSs will improve when GWAS sample sizes have increased by orders of magnitude, ancestral diversity in genetic studies expands, and when more risk loci are detected. However, realizing this potential will require significant investment, international collaboration, and validation. In the interim, family history of STBs – and to a lesser extent MDD – can help identify individuals who may benefit from preventive interventions. Also, rather than viewing genetic liability as fixed, it is important to recognize that genetic factors can not only vary across the lifespan (Neale and Cardon 1992) but also influence sensitivity to environmental conditions (Kendler 1998). The genetic and environmental architectures we identified between MDD and STBs illustrate the importance of considering both heritable and environmental contributions to risk, though further research is needed to translate these findings into clinical practice.

Sex differences in suicide-related phenotypes show complex and often contrasting patterns across populations. While our sample showed no significant sex differences, this finding is particularly noteworthy when compared to established national patterns in our study location of Australia. Here, women report higher lifetime prevalence of STBs (18.3%) compared to men (15.0%) (Australian Institute of Health and Welfare 2024). However, this pattern contrasts sharply with suicide completion rates, where men die by suicide at significantly higher rates (18.0 per 100,000) compared to women (5.8 per 100,000) (Australian Institute of Health and Welfare 2024). These opposing patterns between suicidal thoughts and completion rates illustrate the complex nature of sex differences in suicide-related phenotypes within even a single population.

Similar complexities in sex differences are observed in the United States. While traditional national survey data have shown comparable rates of suicidal ideation and attempts between adult men and women, with men having higher death rates (Basterfield et al. 2024), recent research reveals an emerging trend of higher recent suicide attempts among adult females (Olfson et al. 2017). The pattern becomes even more distinct in adolescent populations, where females consistently show higher rates of both suicidal ideation and attempts (Auerbach et al. 2019; Nock et al. 2013). This pattern is supported by the Miranda-Mendizabal et al. meta-analysis (2019), which found that women had significantly higher rates of ideation and attempts than men during adolescence and young adulthood. This aligns with the recent findings from Xiao et al. (2021), which showed the prevalence of suicidal ideation was consistently higher among female adolescents compared to males from 1991 to 2019. These age-specific patterns along with the broader population trends, highlight the importance of considering both developmental stage and context when examining sex differences in suicide-related phenotypes. The relationship between sex and suicide risk appears to manifest differently not only across age groups but also between community and clinical samples.

## Limitations

Our results should be interpreted in the context of at least five limitations.

First, while common in studies of STBs, the wide confidence intervals, particularly for suicide-related phenotypes, remain an important limitation. These intervals reflect genuine uncertainty in our parameter estimates and underscore a fundamental challenge in suicide research: the need for very large samples to achieve adequate statistical power, especially given the relatively low prevalence of suicide attempts. Without more precise estimates, we cannot be entirely certain about the magnitude of genetic and environmental influences, even when the overall pattern aligns with previous research. This limitation reinforces the critical importance of continued investment in large-scale studies of STBs.

Second, to maximize power and to generalize our results to the population at large, we jointly analyzed data from males and females. While we included sex as a fixed covariate in all our analyses, this approach only corrected for mean differences and did not account for potential sex differences in the genetic architecture of either PRS, the DSM-IV MDD diagnosis and STBs. The extent to which genetic influences on STBs are sex-invariant remains unclear, and should be the focus of future research where cohorts are adequately sized for sufficient statistical power. While our pooled analysis allowed us to identify patterns generalizable across sexes, it may have obscured some sex-specific patterns. By combining data from men and women and adjusting for sex differences, we aimed to interpret the observed patterns of association that are shared or common across sexes, thus providing a foundation for understanding broader trends in the population.

Third, the MD PRS was based on the Howard et al. (2019) GWAS meta-analysis of broad sense MD, including both clinically ascertained and self-reported diagnoses. It, therefore, remains unclear whether or not a PRS based on clinically diagnosed MDD would yield similar results or maintain the same pattern of weaker prediction compared to the SB PRS. This distinction may affect the interpretation of genetic overlap between MDD and STBs.

Fourth, we were unable to include a PRS for suicidal ideation based on the large Ashley-Koch et al. (2023) GWAS summary statistics, as these were not available at the time of our analysis. This omission may have restricted our ability to explore the genetic contributions specific to SI phenotypes. However, the Ashley-Koch GWAS relied on the Million Veteran Program, a predominantly male (90%) cohort of military veterans, which is not representative of our Brisbane population-based sample of young adult men and women. The impact of this omission is likely to be modest. Given the limited explanatory power of the SB PRS in our study, the complexity of STBs suggests that larger, population-based GWAS discovery samples are needed to develop PRSs that outperform the current PRS.

Finally, both PRSs used in this study likely suffer from weak instrumental bias, a common issue in PRS analyses. The predictive power of these PRSs is expected to improve as discovery GWAS analyses increase in sample size and statistical power. This limitation underscores the need for cautious interpretation of our results and highlights the importance of ongoing efforts to refine and improve genetic risk prediction tools in psychiatric research.

## Conclusion

This study provided compelling evidence for shared genetic architecture between DSM-IV MDD and STBs through complementary twin and molecular genetic analyses. Our multivariate twin analyses revealed remarkably high genetic correlations among STBs (rA = 0.85–0.99) and moderate genetic overlap with MDD (rA = 0.48–0.65). At the molecular level, while both PRSs showed modest prediction of suicide outcomes, the MD PRS demonstrated more consistent prediction of MDD compared to the variable prediction by the SB PRS. These findings suggest partially distinct genetic architectures underlying MDD and STBs, highlighting the importance of developing better molecular predictors of suicide risk. The substantial non-shared environmental influences and weak environmental correlations between MDD and STBs indicate distinct environmental pathways to each outcome, underscoring the importance of identifying specific environmental risk factors. While current PRSs have limited clinical utility, family history of STBs may help identify individuals needing preventive intervention. Future research should focus on larger, more diverse samples to improve genetic prediction, while exploring how genetic risk factors interact with environmental exposures across the lifespan.

## Supplementary Information

Below is the link to the electronic supplementary material.

Supplementary Material 1

## Data Availability

No datasets were generated or analysed during the current study.
