# Supplementary material for: Genetic Overlap Between DSM-IV Major Depressive Disorder and Suicidal Behaviors: Evidence from Polygenic Risk Scores in Young Adult Twins
Source: Behav Genet. 2025 Sep 24;55(6):423–37. doi: 10.1007/s10519-025-10234-0 (PMC12719343; doi:10.1007/s10519-025-10234-0)
Supplement: Supplementary file 1 — Supplementary Material 1 [file 10519_2025_10234_MOESM1_ESM.docx]

**Supplementary Methods: Assessment of Threshold Homogeneity and Familial Aggregation**

Prior to modeling, we evaluated the assumption of threshold homogeneity and preliminarily assessed sources of familial aggregation. Each observed phenotypic variable was assessed across four distinct measurement sources: MZ twin 1, MZ twin 2, DZ twin 1, and DZ twin 2. We tested three models with increasing constraints:

1. Unconstrained Model (Null Model)
   - No constraints applied
   - Serves as a baseline for comparison
2. Threshold Homogeneity Model
   - Constrains thresholds to be equal across all four groups
   - Tests the assumption that item or diagnostic thresholds are equivalent across all measurement instances
3. Equal Correlation Model
   - Builds on the Threshold Homogeneity Model
   - Additionally constrains MZ and DZ twin pair correlations to be equal
   - Provides a preliminary determination of significant familial aggregation and potential sources thereof

See Supplementary Table S3 for the model fitting comparisons.

Comparing the Unconstrained Model with the Threshold Homogeneity Model tests if thresholds can be constrained across groups. The Equal Correlation Model examines if MZ and DZ twin pair correlations can be equalized. This approach assesses threshold validity and provides insights into genetic and environmental influences on the trait. Differences in MZ and DZ correlations indicate relative contributions of genetic and shared environmental factors. This evaluation ensures validity for subsequent twin modeling analyses.

**Supplementary Figure S1.** Theoretical univariate twin model for decomposing genetic and environmental influences on suicide behaviors.

Note: A = additive genetic influences; C = shared/common environmental influences; E = non-shared/unique environmental influences (including measurement error). MZ = monozygotic twins; DZ = dizygotic twins. Twin modeling assumes: (1) MZ twins share 100% of their genes while DZ twins share on average 50% (raMZ = 1.0, raDZ = 0.5); (2) shared environmental effects are equal in MZ and DZ twin pairs (rC = 1.0); (3) non-shared environmental effects (E) are by definition uncorrelated. Model identification is achieved by constraining the factor loadings to one. The relative contribution of genetic and environmental influences is estimated through the variances (double-headed arrows) of the latent A, C, and E factors.

**Supplementary Figure S2**. Probabiltiy of endorsing suicide behaviors and DSM-IV MDD diagnosis by age plots

**Supplementary Methods: Bootstrap analysis of heritability estimates**

We fitted a bivariate twin model to MDD and suicide attempt to evaluate the stability of heritability estimate of the latter. Full model fitting comparisons are shown in Supplementary Table S6. We conducted a non-parametric bootstrap analysis using the best-fitting bivariate AE model. We performed 1000 bootstrap iterations, resampling MZ and DZ twin pairs separately with replacement to maintain the original twin structure. For each bootstrap sample, we refit the complete bivariate AE model, maintaining all original model specifications including threshold adjustments for sex effects on MDD and age effects on suicide attempt.

The bootstrap analysis yielded heritability estimates of 40% (95% CI: 24-56%, SD = 0.084) for MDD and 74% (95% CI: 20-98%, SD = 0.255) for suicide attempt. As shown in Figure S3, the distribution of bootstrap estimates for MDD showed approximately normal distribution, while suicide attempt estimates showed far greater dispersion.

While the bootstrap point estimates align with our primary analysis, the wider confidence intervals for suicide attempt heritability suggest greater uncertainty. This increased uncertainty likely reflects the statistical challenges of estimating genetic influences on less prevalent phenotypes. Our results underscore the need for cautious interpretation of heritability estimates for rare events, even when point estimates indicate substantial genetic influence.

**Supplementary Figure S3**. Bootstrap distributions of heritability estimates for Major Depressive Disorder (A1) and suicide attempt (A2)

Note: The figure shows frequency distributions from 1000 bootstrap iterations of heritability estimates from a bivariate AE twin model. Panel A shows the distribution of heritability estimates for Major Depressive Disorder (A1). Panel B shows the distribution of heritability estimates for suicide attempt (A2).
